# Chloroquine triggers Epstein-Barr virus replication through phosphorylation of KAP1/TRIM28 in Burkitt lymphoma cells

**DOI:** 10.1371/journal.ppat.1006249

**Published:** 2017-03-01

**Authors:** Xiaofan Li, Eric M. Burton, Sumita Bhaduri-McIntosh

**Affiliations:** 1 Division of Infectious Diseases, Department of Pediatrics, Stony Brook University School of Medicine, Stony Brook, NY, United States of America; 2 Department of Molecular Genetics and Microbiology, Stony Brook University, Stony Brook, NY, United States of America; Wistar Institute, UNITED STATES

## Abstract

Trials to reintroduce chloroquine into regions of Africa where *P*. *falciparum* has regained susceptibility to chloroquine are underway. However, there are long-standing concerns about whether chloroquine increases lytic-replication of Epstein-Barr virus (EBV), thereby contributing to the development of endemic Burkitt lymphoma. We report that chloroquine indeed drives EBV replication by linking the DNA repair machinery to chromatin remodeling-mediated transcriptional repression. Specifically, chloroquine utilizes ataxia telangiectasia mutated (ATM) to phosphorylate the universal transcriptional corepressor Krüppel-associated Box-associated protein 1/tripartite motif-containing protein 28 (KAP1/TRIM28) at serine 824 –a mechanism that typically facilitates repair of double-strand breaks in heterochromatin, to instead activate EBV. Notably, activation of ATM occurs in the absence of detectable DNA damage. These findings i) clarify chloroquine’s effect on EBV replication, ii) should energize field investigations into the connection between chloroquine and endemic Burkitt lymphoma and iii) provide a unique context in which ATM modifies KAP1 to regulate persistence of a herpesvirus in humans.

## Author summary

Viruses that persist for the life of the host, like the herpesvirus Epstein-Barr virus (EBV), tightly regulate lytic replication to reduce killing of host cells and ensure virus survival. We show that repression of EBV replication is disrupted by the antimalarial drug chloroquine which modifies an otherwise normal cellular mechanism that repairs DNA, to influence gene expression through a process known as chromatin remodeling. This finding a) reveals a new connection between the DNA repair machinery and gene regulation and b) resolves a long-standing dispute over whether chloroquine increases EBV replication, thereby contributing to endemic Burkitt lymphoma, a cancer almost uniformly associated with EBV. There are ongoing efforts to re-introduce chloroquine into parts of Africa where falciparum malaria has regained susceptibility to chloroquine.

## Introduction

Two earlier studies reported contradictory findings on the ability of chloroquine to lytically (re)activate Epstein-Barr virus (EBV) in human B lymphocytes [1,2]. This left open the debate on whether chloroquine might contribute to the high rates of endemic Burkitt lymphoma (eBL) in malaria holoendemic areas of Africa. eBL is almost uniformly associated with EBV and is thought to arise from germinal center B cells harboring clonal EBV in every cell of the tumor [3]. While we did not set out to address the possibility of a link between chloroquine and EBV lytic replication, our investigations into the property of partial permissiveness of EBV [4,5], a member of the herpesvirus family and a WHO group I carcinogen, reveal that chloroquine activates EBV lytic cycle in eBLs.

A key feature of herpesviruses is the ability to restrict the number of latently/quiescently infected cells that respond to lytic triggers by producing infectious virions. This property of partial permissiveness limits virus-mediated pathology while ensuring persistence in the cell [4–6]. In the case of EBV, this property also curbs approaches to effectively activate the virus into the lytic phase to kill cancers bearing EBV. Our efforts to reveal strategies to enhance lytic susceptibility of EBV have focused on identifying regulatory mechanisms of lytic susceptibility that are shared by members of the herpesvirus family. We previously reported that the transcription factor signal transducer and activator of transcription 3 (STAT3) plays a key role in regulating susceptibility of both oncogenic human herpesviruses EBV and Kaposi’s Sarcoma Associated Herpesvirus (KSHV) to lytic signals [4,5,7]. For KSHV, STAT3 functions via the universal transcriptional co-repressor Krüppel-associated Box (KRAB)-associated protein (KAP)-1 [7]–prompting us to investigate the contribution of KAP1/tripartite motif protein 28 (TRIM28) towards lytic susceptibility of EBV.

KAP1’s ability to remodel chromatin is primarily regulated by post-translational modifications. KAP1 harbors an E3 ligase activity for Small Ubiquitin-like Modifier (SUMO) protein and is subject to constitutive SUMOylation within KAP1 oligomers. SUMOylation creates binding sites on KAP1 for two histone modifiers (CHD3 and SETDB1) that mediate histone deacetylation and trimethylation at lysine 9 of histone 3 (H3K9) respectively, consequently causing chromatin condensation and transcriptional repression [8,9]. Phosphorylation of KAP1 at S824 impairs SUMOylation of KAP1 and antagonizes its ability to condense chromatin. A key component of the DNA damage response triggered by double-strand DNA breaks, particularly in the context of heterochromatin, is phosphorylation of KAP1 at S824 resulting in remodeling, relaxation and repair of damaged DNA [10]. Although generally thought to be mediated via the PI3-kinase-related kinase ataxia telangiectasia mutated (ATM) [11–13], whether ATM phosphorylates KAP1 or functions via an intermediate kinase is not clear.

We now report that the cellular strategy of KAP1-mediated chromatin remodeling to repair DNA breaks in heterochromatin is hijacked by a ubiquitous cancer-causing virus to derepress viral chromatin, thereby regulating the balance between virus replication and persistence in the host. We also provide novel evidence for direct in situ interaction between endogenous ATM and KAP1 resulting in phosphorylation of KAP1 in lytic cells, even in the absence of observable DNA damage. Importantly, we demonstrate that the antimalarial agent chloroquine utilizes the above strategy to trigger EBV replication, thus resolving the controversy over whether chloroquine increases EBV replication. Its ability to expel EBV from latently infected B cells also makes chloroquine an attractive candidate to directly kill and elicit immune-mediated killing of tumor cells.

## Results

### Manipulation of KAP1 levels modulates EBV lytic activation

**Fig 1:**
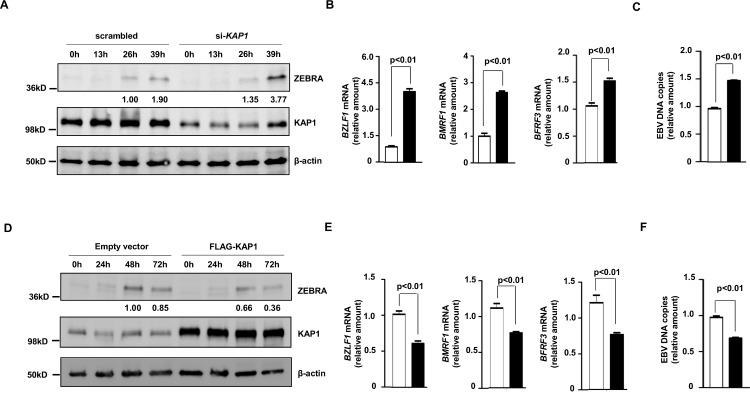
Cellular KAP1 regulates EBV lytic cycle in Burkitt Lymphoma cells. **A-C.** HH514-16 Burkitt lymphoma (BL) cells were transfected with scrambled siRNA (non-targeting control; open bars in B, C) or siRNA to *KAP1* (black bars in B, C); **D-F.** BL cells were transfected with empty vector (open bars in E, F) or pFLAG-CMV2-KAP1 (black bars in E, F). Transfected cells were treated with NaB 24 hours after transfection, and harvested at indicated times (A, D), 24 hours (B, E), or 48 hours (C, F) post-treatment to determine relative amounts of ZEBRA, KAP1 and β-actin by immunoblotting (A, D), relative levels of transcripts from EBV lytic genes *BZLF1*, *BMRF1* and *BFRF3* by qRT-PCR after normalization to 18S rRNA using the ΔΔC_T_ method (B, E), and relative levels of cell-associated EBV DNA by qPCR (C, F). Error bars: SEM of 3 experiments with 4 technical replicates. Western blots are representative of 2 experiments. Numbers below blots indicate relative amounts of protein after normalization to β-actin.

We first tested the effect of manipulating KAP1 on the EBV lytic cycle using HH514-16 eBL cells in which EBV is tightly latent but can be switched into the lytic phase using exogenous triggers [4–6]. Compared to scrambled siRNA-transfected cells, knockdown of KAP1 with two different siRNAs accompanied by exposure to the lytic cycle inducing agent sodium butyrate (NaB; an HDAC inhibitor) resulted in elevated levels of the EBV latency-to-lytic switch protein ZEBRA (*BZLF1* product), transcripts from viral lytic genes *BZLF1* (immediate early lytic gene), *BMRF1* (early lytic gene), and *BFRF3* (late lytic gene), and EBV load (Fig 1A–1C and S1 Fig). In contrast, compared to empty vector-transfected cells, overexpression of KAP1 suppressed the levels of ZEBRA, transcripts from *BZLF1*, *BMRF1*, and *BFRF3*, and EBV DNA copy number (Fig 1D–1F). As expected, knockdown and overexpression of KAP1 were observed in Fig 1A and 1D, respectively. Thus, cellular KAP1 functions as a regulator of EBV lytic activation.

### Phosphorylation at Serine 824, induced by lytic triggers, impairs KAP1’s ability to curtail EBV replication

**Fig 2:**
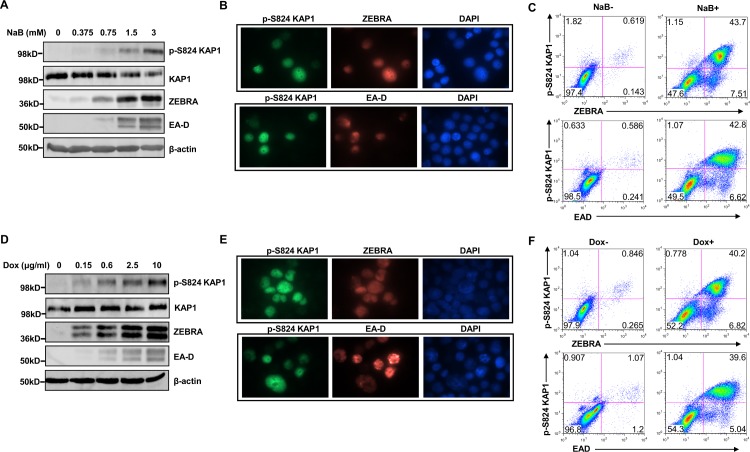
KAP1 is phosphorylated at serine 824 during EBV lytic cycle in Burkitt lymphoma cells. **A, D.** HH514-16 BL cells and HH514-16-derived CLIX-FZ cells were treated with increasing doses of NaB and doxycycline respectively for 24 hours, and lysates from cells were analyzed via immunoblotting with antibodies as indicated. **B, C, E, F.** HH514-16 cells were treated with NaB (B, C) or CLIX-FZ cells were treated with doxycycline (E, F) for 24 hours and stained with either anti-phospho KAP1 (S824) plus anti-ZEBRA antibodies (upper panels) or anti-phospho KAP1 (S824) plus anti-EA-D antibodies (lower panels) and visualized at 1000X magnification (B, E) or subjected to flow cytometry (C, F).

Since KAP1 is able to regulate EBV lytic susceptibility and since the transcriptional repressive function of KAP1 is regulated by post-translational modifications, most commonly phosphorylation at S824 impairing KAP1’s ability to repress target genes [9], we examined whether KAP1 underwent phosphorylation at S824 upon lytic activation. Exposure of HH514-16 cells to NaB resulted in phosphorylation of KAP1 at S824, but only in lytic, i.e. ZEBRA^+^ and EA-D^+^ cells; refractory cells did not demonstrate p-KAP1 (Fig 2A–2C). Importantly, treatment of BJAB cells, an EBV-negative B lymphoma cell line, with NaB did not induce phosphorylation of KAP1, whereas exposure to the DNA damaging agent etoposide did (S2 Fig). As an alternative to a chemical lytic trigger, we activated the lytic cycle by inducing expression of ZEBRA in HH514-16-derived CLIX-FZ cells that contain a stably-integrated doxycycline-inducible tagged *BZLF1* gene, and detected phosphorylation of KAP1 at S824, again only in lytic cells (Fig 2D–2F). Notably, spontaneous lytic cells also stained for p-KAP1 while latent cells did not (Fig 2C and 2F).

**Fig 3:**
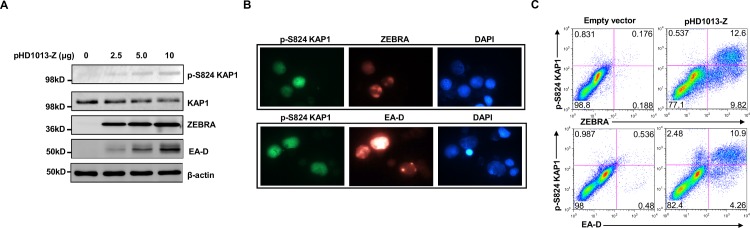
Phosphorylation of KAP1 at serine 824 is induced during EBV lytic cycle in lymphoblastoid cells. **A.** Lymphoblastoid cells were transfected with indicated amounts of ZEBRA-expressing plasmid (pHD1013-Z), with a constant amount of total transfected DNA using empty vector. Transfected cells were harvested 24 hours later and subjected to immunoblotting with antibodies as indicated. **B, C.** Lymphoblastoid cells were transfected with pHD1013-Z plasmid, harvested at 24 hours and stained with either anti-phospho KAP1 (S824) plus anti-ZEBRA antibodies (upper panels) or anti-phospho KAP1 (S824) plus anti-EA-D antibodies (lower panels) and visualized at 1000X magnification (B) or subjected to flow cytometry (C).

In a parallel set of experiments, we examined the effects of lytic activation in an EBV-transformed lymphoblastoid cell line (LCL). LCLs are generally resistant to commonly used lytic activation triggers but can be induced to support the EBV lytic cycle following introduction of the *BZLF1* gene. Consistent with our findings with NaB-treated HH514-16 cells, we found increased phosphorylation of KAP1 at S824, again primarily in ZEBRA^+^ and EA-D^+^ (lytic) LCLs following transfection with *BZLF1* plasmid; very few refractory cells demonstrated pKAP1 (Fig 3).

**Fig 4:**
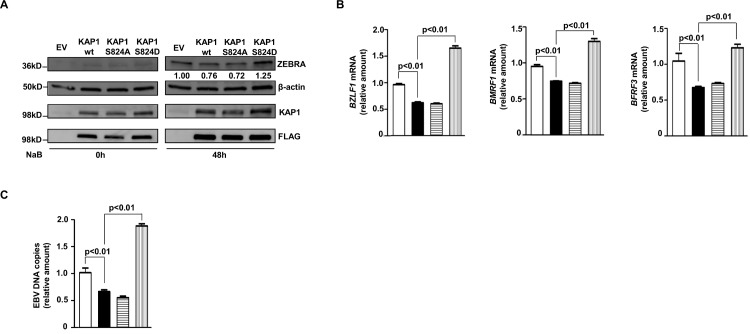
Phosphorylation of KAP1 at serine 824 impairs its ability to restrain EBV lytic cycle. HH514-16 BL cells were transfected with empty vector (EV in A; open bar in B and C), pFLAG-CMV2-KAP1 (KAP1 wt in A; black bar in B and C), pFLAG-CMV2-KAP1-S824A (KAP1 S824A in A; bars with horizontal lines in B and C) or pFLAG-CMV2-S824D (KAP1 S824D in A; bars with vertical lines in B and C), treated with NaB after 24 hours, and harvested immediately (0h) or after 48 hours for immunoblotting with antibodies as indicated (A), harvested after 24 hours for qRT-PCR to determine the relative levels of transcripts from EBV lytic genes *BZLF1*, *BMRF1* and *BFRF3* (B) or harvested after 48 hours to compare the relative levels of cell-associated EBV DNA by qPCR (C). Experiments were performed at least twice; error bars for B and C represent 3 technical replicates from 2 experiments.

To investigate the contribution of the S824 residue towards latency-to-lytic cycle transition, we introduced wild-type versus mutant KAP1 (S824A [phospho-dead] and S824D [phospho-mimetic]) in HH514-16 cells. As expected, compared to empty vector-transfected cells, FLAG-KAP1-wt and S824A KAP1 each repressed ZEBRA protein, EBV lytic transcripts and EBV DNA copy number. In contrast, compared to FLAG-KAP1-wt, S824D KAP1 resulted in increased ZEBRA protein, EBV lytic transcripts and EBV DNA copy number (Fig 4A–4C). Thus, lytic trigger-induced phosphorylation of KAP1 at S824 impairs KAP1’s ability to restrict EBV replication.

### ATM causes phosphorylation of KAP1 at S824 exclusively in lytic cells

**Fig 5:**
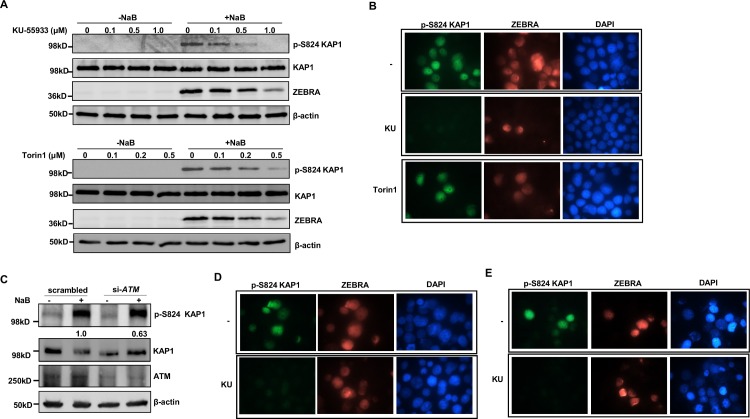
ATM induces phosphorylation of KAP1 at S824 upon exposure to lytic trigger. **A.** HH514-16 BL cells were treated with increasing amounts of PI3 kinase-related kinase inhibitors (KU-55933 or Torin1; left panels) or PI3KK inhibitors plus NaB (right panels). After 24 hours, cells were harvested and lysates analyzed via immunoblotting with the antibodies indicated. **B.** HH514-16 cells were treated with NaB (upper panel) or NaB plus KU-55933 (1μM; middle panel) or NaB plus Torin1 (0.5μM; lower panel) for 24 hours and stained with anti-phospho KAP1 (S824) plus anti-ZEBRA antibodies and visualized at 1000X magnification. **C.** HH514-16 cells were transfected with scrambled siRNA or siRNA to *ATM*, treated with NaB after 24 hours and harvested after another 24 hours. Cell lysates were analyzed by immunoblotting with the antibodies indicated. Numbers below p-S824 KAP1 blot indicate relative amounts of pKAP1 after normalization to total KAP1. **D.** HH514-16-derived CLIX-FZ cells were exposed to doxycycline (upper panel) or doxycycline plus KU-55933 (lower panel) for 24 hours and stained with anti-p-S824 KAP1 plus anti-ZEBRA antibodies and visualized at 1000X magnification. **E.** Lymphoblastoid cells were transfected with pHD1013-Z plasmid and simultaneously treated with vehicle (-KU) or KU-55933. Treated cells were harvested 24 hours later and subjected to staining with anti-phospho KAP1 (S824) plus anti-ZEBRA antibodies and visualized at 1000X magnification. Experiments were performed twice.

To determine how KAP1 is phosphorylated during EBV lytic activation, we turned to the phosphatidylinositol 3-kinase-related kinase (PIKK) ataxia telangiectasia mutated (ATM). A key event triggered by double-strand DNA breaks, particularly in the context of heterochromatin, is ATM-mediated phosphorylation of KAP1 at S824 which facilitates repair of damaged DNA [10–13]. We found that the presence of the ATM inhibitor KU-55933 during lytic activation resulted in impaired p-S824 KAP1 levels without affecting total KAP1 levels; simultaneously, the level of ZEBRA protein and the number of lytic cells were suppressed (Fig 5A and 5B). Importantly, in cells with ZEBRA expression, treatment with KU-55933 completely abolished phosphorylation of KAP1 (Fig 5B), indicating that ATM is required both for EBV lytic gene expression and phosphorylation of KAP1 at S824. In contrast, the mTOR (another PIKK) inhibitor Torin1 impaired lytic susceptibility without affecting p-S824 KAP1 levels in lytic cells (Fig 5A and 5B); of note, inhibitors were used at the lowest concentrations at which there was discernible reduction in p-S824 KAP1 levels. In a complementary approach, we found that compared to scrambled siRNA-transfected cells, the level of p-S824 KAP1 decreased by 40% in cells transfected with siRNA to *ATM* (Fig 5C). As expected, si-*ATM* transfected cells demonstrated lower amounts of ATM.

Since lytic activation/ZEBRA expression results in phosphorylation of KAP1 (Figs 2 and 3) and both ZEBRA expression and KAP1 phosphorylation require ATM (Fig 5A–5C), we asked if ATM is also functionally interposed between ZEBRA and KAP1. We therefore expressed ZEBRA in BL cells and LCLs while simultaneously treating with KU-55933. We found that ZEBRA-expressing cells demonstrated substantially reduced amounts of p-S824 KAP1 in the presence of KU-55933 compared to solvent-treated cells (Fig 5D and 5E), indicating that functionally, ATM could be placed downstream of ZEBRA but upstream of KAP1; moreover, KAP1 phosphorylation is dependent on ATM. Taken together, these results indicate that lytic activation/ZEBRA expression leads to ATM-mediated phosphorylation of KAP1 at S824.

### Endogenous ATM and KAP1 interact directly only in lytic cells

**Fig 6:**
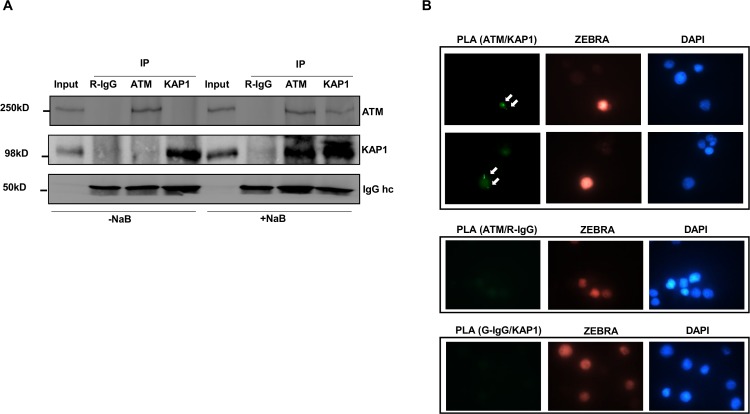
Endogenous ATM and KAP1 directly interact in situ exclusively in lytic cells. **A.** HH514-16 cells were treated with NaB or left untreated and harvested 24 hours later for immunoprecipitation with normal rabbit IgG, rabbit anti-ATM or rabbit anti-KAP1 antibody. Input samples and immunoprecipitated materials were subjected to immunoblotting with indicated antibodies; IgG hc: IgG heavy chain. **B.** HH514-16 cells were treated with NaB and harvested at 24 hour post-treatment for examination by proximity ligation assay (PLA) with antibodies to ATM and KAP1 (upper 2 panels), anti-ATM antibody and rabbit IgG (middle panel) or anti-KAP1 antibody and goat IgG (lower panel) and subsequent immunostaining with antibody against ZEBRA; arrows: fluorescent foci indicative of intermolecular interactions. Experiments were performed twice.

Following DNA double-strand breaks in heterochromatin DNA, ATM induces phosphorylation of KAP1 [11–13]; however, whether ATM interacts with KAP1 or functions via intermediary kinases remains unclear. We found that compared to latently infected cells (NaB-untreated), an antibody to ATM was able to co-precipitate KAP1 in cells exposed to the lytic trigger NaB; similarly, an antibody to KAP1 co-precipitated ATM in the same cells (Fig 6A). We addressed whether endogenous ATM and KAP1 interact in situ in NaB-treated BL cells using proximity ligation assay (PLA). Fig 6B shows that ATM interacted with KAP1 only in lytic cells, as seen by small spots of fluorescence; ATM-KAP1 interaction was not observed in refractory cells. PLA signal is typically absent if binding partners are more than 40 nanometer apart [14,15], placing ATM and KAP1 in very close proximity. Thus upon activation, ATM directly interacts with KAP1, resulting in p-S824 KAP1 and ultimately latency-to-lytic transition.

### ATM activator chloroquine induces phosphorylation of KAP1 at S824 and EBV lytic replication

**Fig 7:**
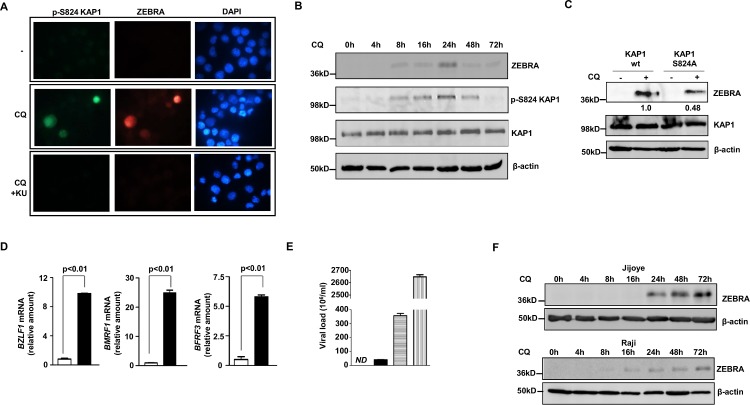
Chloroquine induces phosphorylation of KAP1 at S824 and activates EBV lytic cycle in Burkitt lymphoma cells. **A.** HH514-16 BL cells were treated with chloroquine (CQ) or chloroquine plus KU-55933 (CQ+KU) and harvested at 24 hours followed by staining with anti-phospho KAP1 (S824) plus anti-ZEBRA antibodies and visualized at 1000X magnification. **B.** HH514-16 BL cells were treated with chloroquine and harvested at different times post-treatment for immunoblotting with antibodies as indicated. **C.** HH514-16 cells were transfected with pFLAG-CMV2-KAP1 (wt) or pFLAG-CMV2-KAP1-S824A. After 48 hours, cells were treated with chloroquine or left untreated for another 48 hours and harvested for immunoblotting with indicated as antibodies. Numbers below bands indicate relative amounts of ZEBRA after normalization to β-actin. **D.** HH514-16 cells were treated with chloroquine (black bars) or left untreated (open bars), harvested 24 hours after treatment and relative levels of transcripts from EBV lytic genes *BZLF1*, *BMRF1* and *BFRF3* were determined by qRT-PCR after normalization to 18S rRNA using the ΔΔC_T_ method. **E.** HH514-16 cells were untreated (open bar), treated with chloroquine (CQ; black bar: 10μM; horizontal striped bar: 200μM) or NaB (vertical striped bar), released virus particles were pelleted from supernatant 7 days later, treated with DNase, and quantified using q-PCR; ND: not detectable. **F.** BL cell lines Jijoye and Raji were treated with chloroquine (CQ) and harvested at different times post-treatment for immunoblotting with antibodies as indicated. Error bars for D and E represent 3 technical replicates from 2 experiments.

Chloroquine, a well-characterized antimalarial agent, has been reported to activate ATM [16]. We asked if chloroquine could cause phosphorylation of KAP1 and activate EBV replication. Indeed in BL cells, chloroquine caused phosphorylation of KAP1 at S824 and lytic activation; moreover, pKAP1 was observed strictly in ZEBRA^+^/lytic cells. Importantly, phosphorylation of KAP1 and lytic activation in response to chloroquine were impaired in the presence of the ATM inhibitor KU-55933 (Fig 7A). Chloroquine increased phosphorylation of KAP1 and expression of ZEBRA over time (Fig 7B). Furthermore, expression of ZEBRA in response to chloroquine depended on phosphorylation of KAP1 at S824 as demonstrated by a drop in the ZEBRA level following overexpression of the phospho-dead mutant (S824A) of KAP1 compared to wt KAP1 (Fig 7C).

Treatment with chloroquine increased levels of EBV lytic transcripts as well as release of encapsidated EBV particles (Fig 7D and 7E); the latter indicates that chloroquine-activated EBV lytic cycle reaches completion and virus particles are released from lytic cells. While NaB clearly was more robust than chloroquine in its ability to increase the extracellular viral load, chloroquine caused a substantial increase in the number of released viral particles even at a concentration of 10μM. We also tested the effect of chloroquine on two other eBL cell lines, Jijoye and Raji, and found that chloroquine induced the expression of ZEBRA in both cell lines, although with different temporal patterns (Fig 7F). Thus, chloroquine activates EBV replication in BL cell lines through ATM and KAP1.

**Fig 8:**
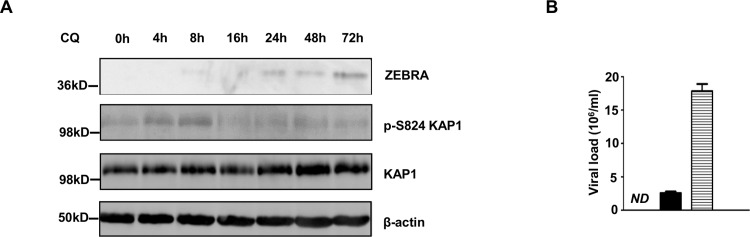
Chloroquine induces phosphorylation of KAP1 at S824 and activates EBV lytic cycle in lymphoblastoid cells. **A.** Lymphoblastoid cells were treated with chloroquine (CQ) and harvested at different times post-treatment for immunoblotting with antibodies as indicated. **B.** Lymphoblastoid cells were untreated (open bar), treated with 10μM CQ (black bar) or 100μM CQ (horizontal striped bar) every 3 days, released virus particles were pelleted from supernatant 9 days later, treated with DNase and quantified using q-PCR. ND: not detectable; error bars represent 3 technical replicates from 2 experiments.

Similar to BL cell lines, exposure of LCLs to chloroquine resulted in phosphorylation of KAP1 at S824 and ZEBRA expression (Fig 8A). Interestingly, pKAP1 was observed earlier in LCLs compared to HH514-16 cells treated with chloroquine (Fig 8A versus Fig 7B). Also consistent with our observation in chloroquine-treated HH514-16 cells, the EBV lytic cycle reached completion in LCLs, again releasing substantial numbers of viral particles into the supernatant, even at the 10μM concentration of chloroquine (Fig 8B). At 10μM concentration, a serum level that results from medically relevant doses of chloroquine used for malaria treatment, HH514-16 cells released about 10-fold more virus particles compared to LCLs (Fig 7E versus Fig 8B). Thus, chloroquine via ATM induces phosphorylation of KAP1 at S824, thereby triggering the complete EBV lytic cycle in BL cells and LCLs.

### Chloroquine and NaB activate ATM in the absence of observable DNA damage

**Fig 9:**
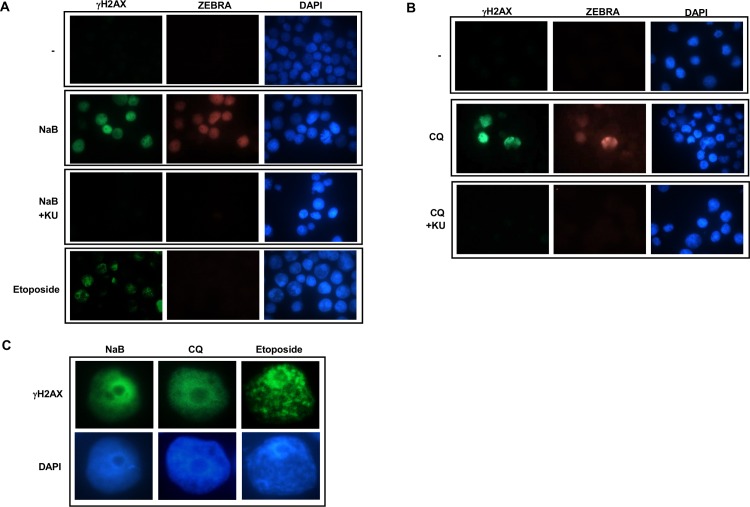
NaB and chloroquine induce EBV replication without observable DNA damage. **A.** HH514-16 cells were left untreated, treated with NaB or NaB plus KU-55933 for 24 hours or with etoposide for 16 hours and stained with anti-γH2AX plus anti-ZEBRA antibodies and visualized at 1000X magnification. **B.** HH514-16 BL cells were treated with chloroquine or chloroquine plus KU-55933 and harvested at 24 hours after treatment, stained for γH2AX and ZEBRA and visualized at 1000X magnification. **C.** Enlarged representative images of cells treated with NaB, chloroquine or etoposide. Experiments were performed twice.

DNA damage, primarily double-strand breaks, activate ATM [17]. In examining whether ATM activation during EBV lytic cycle was associated with DNA double-strand breaks, we undertook staining for γH2AX, a substrate of ATM and a marker of DNA damage. We observed increased amounts of γH2AX only in lytic cells; however, instead of discrete foci indicative of areas of damaged DNA, the staining pattern appeared diffuse. In contrast, cells exposed to etoposide, a double-strand break inducing agent, showed γH2AX foci as expected, but no lytic activation (Fig 9A and 9C). Importantly, the presence of KU-55933 during lytic activation resulted in loss of both lytic cells and γH2AX staining. Consistent with ATM activation, treatment with chloroquine also resulted in increased level of γH2AX in lytic cells that was inhibited in the presence of KU-55933. Again, nuclear γH2AX staining was diffuse, consistent with lack of DNA damage (Fig 9B and 9C). Notably, chloroquine treatment resulted in approximately 14–15% lytic cells (Figs 7A and 9B). Thus, in response to lytic trigger or chloroquine, ATM is activated despite a lack of observable DNA damage; ATM then interacts with KAP1 to cause phosphorylation at S824 resulting in derepression of lytic genes.

**Fig 10:**
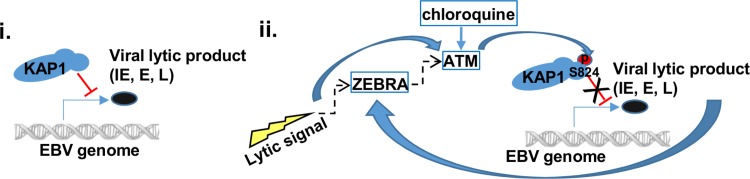
Model of ATM-KAP1-mediated regulation of EBV replication. i. KAP1 represses EBV lytic genes in latently infected cells. ii. Lytic cycle activation signals cause ATM-mediated phosphorylation of KAP1 at S824; phosphorylated KAP1 is unable to repress EBV lytic genes. These events allow expression of lytic genes of all kinetic classes in response to lytic cycle activation triggers. Events triggering the initial expression of ZEBRA and activation of ATM are unclear and shown by dashed arrows. Chloroquine, known to activate ATM, causes phosphorylation of KAP1 at S824 resulting in activation of EBV lytic cycle; IE: immediate early, E: early, L: late lytic genes.

Taken together, our findings support a model in which KAP1, un-phosphorylated at S824, represses lytic genes in latently infected cells (Fig 10-i). Lytic signals or ZEBRA cause ATM to phosphorylate KAP1 at S824; this modification renders KAP1 unable to repress EBV lytic genes, thereby resulting in expression of lytic genes of all kinetic classes (Fig 10-ii). Chloroquine, by activating ATM, feeds into this pathway to trigger EBV lytic cycle and evicts the virus.

## Discussion

Our work demonstrates that EBV has appropriated components of the DNA repair machinery typically operational at heterochromatin DNA to regulate the balance between lytic (re)activation and persistence. While ATM has previously been shown to be required for efficient EBV lytic activation [18–20], the precise mechanism and the substrate for its kinase activity were not known. We also demonstrate that endogenous ATM and KAP1 interact in situ resulting in phosphorylation of KAP1. Notably, the signal that activates ATM during EBV lytic cycle does not require DNA double-strand breaks. Furthermore, DNA damage-mediated ATM activation does not necessarily induce EBV replication; either additional triggers are needed to activate transcription of viral genes or damage to viral DNA cripples lytic replication.

In the setting of EBV lytic replication, we postulate that ATM may be activated by: i) The EBV protein kinase, known to be expressed downstream of ZEBRA; lack of DNA damage foci is consistent with this option. ii) Distortion of DNA caused by binding of ZEBRA, both a replication protein and a transactivator [21], may be detected as DNA “lesions” leading to ATM activation; loss of ATM activation following transfection of DNA binding domain mutants of ZEBRA [20] supports options i and ii. iii) Low levels of DNA damage that may escape detection by conventional means or specific types of DNA lesions may activate ATM; activation of EBV lytic cycle by DNA crosslinking agents supports the latter possibility [18].

Modification of the S824 residue of KAP1 represents a shared mechanism for regulating persistence versus lytic cycle activation in at least three members of the herpesvirus family; yet the responsible kinases are distinct: viral protein kinase in KSHV [22], mTOR in HCMV [23] and ATM in EBV. KAP1 also participates during initial infection of KSHV, establishment of HCMV latency, and maintenance of EBV and KSHV latency [23–26]. How KAP1 is recruited to viral DNA remains an open question for both HCMV and EBV although in KSHV-infected cells, cellular Nrf2 and the viral protein LANA recruit KAP1 to viral DNA [26,27]. Since KAP1 is not a bonafide DNA binding protein, we suspect that it is recruited to specific sites on the viral genome via other proteins, with Krüppel associated box (KRAB)-zinc finger proteins being the most likely candidates [9].

Two recent studies have reported on the effect of chloroquine on lytic (re)activation of herpesviruses. The first study, consistent with our observation, found that the lytic cycle of HCMV was activated, albeit after 3 doses of chloroquine [23]. The second, found that chloroquine hindered virion production of KSHV and EBV in response to lytic cycle inducing agents [28]. Although in apparent contradiction to our findings, the latter study did not examine the effect of chloroquine alone beyond 6 hours; further, this study focused on the effect of chloroquine in the setting of known lytic cycle inducing agents.

A multitude of studies have causally linked malaria to eBL. Malaria causes polyclonal activation of B cells resulting in their proliferation and differentiation [29]. Proliferation increases the number of B cells latently infected with EBV while differentiation results in antibody-producing plasma cells. Differentiation into plasma cells triggers lytic activation of EBV [30]. While such EBV lytic activity would normally be curtailed by T cells, malaria also suppresses EBV-directed T cell responses [31–33]; as a result, EBV lytic replication goes unchecked, ultimately resulting in an expansion of the latently infected B cell pool. The link between EBV lytic cycle and eBL development is further strengthened by the following observations: i) increases in serum IgG antibodies to EBV lytic proteins preceded the development of eBL [34] and ii) the sap of the milk bush found more often at the homes of eBL patients in Malawi and known to contain the parent compound of the EBV lytic cycle activating agent phorbol myristic acid, induced c-Myc translocations characteristic of BL in cord blood B cells simultaneously exposed to EBV [35,36]. Our discovery that chloroquine (re)activates EBV supports the idea that chloroquine may contribute to the development of eBL.

A study in Tanzania from 1977 to 1982 reported a decrease in the incidence of eBL during the early years of chloroquine prophylaxis for malaria, followed by its resurgence after completion of the study [37]. This seeming disagreement with our findings, while difficult to resolve in the absence of virologic and immunologic data from the said study, may be fallacious because the decline in eBL incidence had started several years before chloroquine distribution among study subjects began [37]. Further, we postulate that the observed drop in eBL incidence was due to decreased malaria burden resulting in decline in pathologic B cell activation with associated improvement in EBV-directed T cell function. These effects would promote more physiologic numbers of latently infected cells and destroy B cells undergoing EBV lytic cycle activation. In contrast, the rebound in eBL incidence soon after the study could be attributed to the combined effects of increased malaria burden and associated impairment of T cell responses to EBV resulting from the observed emergence of chloroquine resistance during the study together with chloroquine-mediated EBV lytic activation. In a more recent study, chloroquine prevented the development of Myc-induced lymphomagenesis in a mouse model by inhibiting autophagy [38]; however, this experimental system is independent of EBV-infection and therefore models sporadic BL but not eBL.

Our findings resolve a long-standing dispute by confirming the contribution of chloroquine to the EBV lytic cycle, and provide the mechanism. This knowledge is timely especially in light of ongoing efforts to re-introduce chloroquine into parts of Africa where *P*. *falciparum* has regained susceptibility to chloroquine. It is also notable that although chloroquine is no longer prescribed for *P*. *falciparum* malaria in most of Africa, over-the-counter use of chloroquine has continued over the last few decades because of its broad anti-pyretic and anti-inflammatory properties [39]. Whether chloroquine-mediated EBV lytic activation contributes to the development of eBL, particularly in the setting of malaria, warrants further investigation. On the other hand, in patients already diagnosed with eBL, chloroquine may be explored in an oncolytic approach to cause lytic EBV-mediated cell death and provoke an anti-tumor immune response. This approach may serve as an alternative to chemotherapy with multiple genotoxic agents, rarely feasible in the low resource settings of equatorial Africa.

## Materials and methods

### Cell culture and chemical treatment

Endemic Burkitt lymphoma cell lines HH514-16 (a gift from Dr. George Miller, Yale University), Jijoye and Raji (gifts from Dr. Janet Hearing, Stony Brook University) were maintained in RPMI 1640 supplemented with 10% fetal bovine serum (Gibco) and 1% penicillin-streptomycin (Gibco). CLIX-FZ (Clone-HH514-16 transfected with pLIX_402-FZ) cells were generated from puromycin (P8833, Sigma-Aldrich) selected HH514-16 cells transfected with pLIX_402-FZ. Lymphoblastoid cell line (LCL) used was generated and maintained as described before [40]. Sodium butyrate (NaB; 3mM; 303410, Sigma-Aldrich), doxycycline (5μg/ml; D9891, Sigma-Aldrich), KU-55933 (1μM; SML1109, Sigma-Aldrich), Torin1 (0.5μM; S2827, Selleckchem) and chloroquine (200μM; C6628, Sigma-Aldrich) were used to treat Burkitt lymphoma cells except where specific concentrations are indicated. KU-55933 (1μM) and chloroquine (100μM) were used to treat lymphoblastoid cells except where specific concentrations are indicated.

### Plasmids, siRNAs and transfection

Plasmid pFLAG-CMV2-KAP1 was a gift from Professor Kum Kum Khanna [41]. Plasmids FLAG-KAP1-S824A and FLAG-KAP1-S824D were generated by replacing the corresponding fragment in pFLAG-CMV2-KAP1 with a product generated from a two-step PCR using pFLAG-CMV2-KAP1 as template and the following primers: forward primer–TCAGGGCTGGAGGTGGTGGCTCCTGAGGGTACC; reverse primers–ACCAGGGCCACCAGACAGCTCCTGGGCACTCAGGCCAGCACCAGGCAGGCT and CTTTAATAAGATCTGGATCTTCAGGGGCCATCACCAGGGCCACCAGACAGCTCCTG were used for construction of pFLAG-KAP1-S824A; forward primer–TCAGGGCTGGAGGTGGTGGCTCCTGAGGGTACC and reverse primers–ACCAGGGCCACCAGACAGCTCCTGGTCACTCAGGCCAGCACCAGGCAGGCT and CTTTAATAAGATCTGGATCTTCAGGGGCCATCACCAGGGCCACCAGACAGCTCCTG were used for construction of pFLAG-KAP1-S824D. Plasmid pHD1013-Z was a gift from Dr. Ayman El-Guindy and described before [42]. Plasmid pLIX_402-FZ (FLAG-*B**Z**LF1*) was constructed by inserting the *BZLF1* coding sequence amplified from a cDNA library of HH514-16 cells with forward primer ATACATCTAGAGCCACCATGGATTACAAGGATGACGACGATAAGATGATGGACCCAAACTCGAC and reverse primer ATACAACCGGTGAAATTTAAGAGATCCTCGTG into pLIX_402 (a gift from David Root [Addgene plasmid # 41394]) at NheI and AgeI sites. BACmid p2089 was a gift from Professor Henri-Jacques Delecluse [43].

siRNAs targeting human KAP1 and ATM transcripts and scrambled/non-targeting siRNA were purchased from Santa Cruz Biotechnology (sc-38550, sc-29761 and sc-37007) and Dharmacon (D-001206-13-05 and M-005046-01) and reconstituted with nuclease free water. HH5514-16 cells and lymphoblastoid cells (1 × 10^6^) were transfected with 20 μg of plasmid or 200 picomoles of siRNA in Ingenio solution (MIR50117, Mirus) using an Amaxa Nucleofector II (program A-024) except where specific amounts of nucleic acids are indicated.

### Antibodies

Antibodies used include rabbit anti-KAP1 Ab (A300-274A, Bethyl Laboratories), rabbit anti-ATM Ab (A300-299A, Bethyl Laboratories), mouse anti-β-actin Ab (AC-15, Sigma), rabbit anti-p-S824-KAP1 Ab (A300-767A, Bethyl Laboratories), mouse anti-EA-D Ab (MAB8186, EMD), mouse anti-FLAG Ab (F3165, Sigma), goat anti-ATM Ab ((A300-136A, Bethyl Laboratories), rabbit anti-p-S139-H2AX (γH2AX) Ab (9718P, Cell Signaling Technology), normal rabbit IgG (sc-2027, Santa Cruz), normal goat IgG (sc-3887, Santa Cruz) and mouse anti-ZEBRA Ab (a gift from Professor Paul Farrell), HRP conjugated goat anti-mouse IgG (H+L) (AP308P, EMD Millipore), HRP conjugated goat anti-rabbit IgG (H+L)(AP307P, EMD Millipore), Fluorescein isothiocyanate (FITC) conjugated donkey anti-rabbit IgG (31568, Thermo Fisher), Phycoerythrin (PE) conjugated goat anti-mouse IgG (sc-3738, Santa Cruz), FITC conjugated goat anti-mouse IgG (F0257, Sigma), and Alexa Fluor 647 conjugated goat anti-rabbit IgG (A-21245, Thermo Fisher).

### Immunoprecipitation and immunoblotting

Sodium butyrate (NaB)-treated or untreated HH514-16 cells in Fig 6A were harvested at 24 hours post treatment with RIPA buffer [50 mM Tris-HCl (pH7.4), 150 mM NaCl, 1% (v/v) NP40, 1% (w/v) deoxycholate, 1 mM EDTA, 1X protease and phosphatase inhibitor cocktail (catalog no. 5872, Cell Signaling Technology)]; 10% of cell lysates was kept as input and the remaining was incubated with indicated antibodies and protein A/G agarose (sc-2003, Santa Cruz Biotechnology) for 16 hours at 4^°^C. Immunoprecipitates were washed with RIPA buffer and subjected to immunoblotting for analysis. Immunoblotting experiments with indicated antibodies were performed as previously described [44].

### Immunofluorescence and flow cytometry

Cells were fixed with BD Cytofix/Cytoperm solution (554722, BD Bioscience) at room temperature for 15 mins, washed with 1X BD Perm/Wash buffer (554723, BD Bioscience) and incubated with indicated primary antibodies for 1 hour at room temperature. After washing, cells were further incubated with corresponding secondary antibodies for another hour at room temperature and then subjected to flow cytometry or mounted using Prolong Gold Antifade with DAPI (4′,6-diamidino-2-phenylindole) (P36935, Thermo Fisher Scientific) for microscopy. Analysis gates for flow cytometry were determined based on parallel staining with isotype-matched control antibodies.

### Proximity ligation assay (PLA)

Proximity ligation assay between ATM and KAP1 protein in sodium butyrate-treated HH514-16 cells was conducted according to manufacturer’s instructions with goat anti-ATM and rabbit anti-KAP1 antibodies (A300-136A and A300-274A, Bethyl Laboratories), Duolink In Situ PLA Probe Anti-Goat MINUS plus Anti-Rabbit PLUS (DUO92006 and DUO92002, Sigma-Aldrich), Duolink In Situ Detection Reagents Green (DUO92014, Sigma-Aldrich) and washing buffers (DUO82049, Sigma-Aldrich).

### Quantitative reverse transcriptase-PCR (qRT-PCR)

qRT-PCRs were performed as previously described [5] and analyzed using the ΔΔC_T_ method [45]; primers sequences included:

forward primer–GTAACCCGTTGAACCCCATT and reverse primer–CCATCCAATCGGTAGTAGCG for *18S rRNA*; forward primer–TTCCACAGCCTGCACCAGTG and reverse primer–GGCAGAAGCCACCTCACGGT for *BZLF1*; forward primer–ACCTGCCGTTGGATCTTAGTG and reverse primer–GGCGTTGTTGGAGTCCTGTG for *BMRF1*; forward primer,–AACCAGAATAATCTCCCCAATG and reverse primer–CGAGGCACCCCAAAAGTC for *BFRF3*.

### Assay for EBV load

Cell-associated EBV DNA was extracted as previously described [7] and viral DNA was quantitated using quantitative-PCR(q-PCR) by amplifying EBV *BALF5* gene with forward primer–CGTCTCATTCCCAAGTGTTTC and reverse primer–GCCCTTTCCATCCTCGTC. In Figs 7 and 8, released EBV particles were pelleted from supernatant, washed with 1X PBS and treated with DNase. Absolute EBV genome copy number was determined with a standard curve obtained through q-PCR using serially diluted BACmid p2089 as template and primers targeting the EBV *BALF5* gene.

### Statistical analysis

*P* values were calculated by comparing the means of two groups of interest using unpaired Student t test.

## Supporting information

S1 Fig Knockdown of endogenous KAP1 enhances EBV lytic activation in Burkitt lymphoma cells. HH514-16 Burkitt lymphoma (BL) cells were transfected with a non-targeting control siRNA (scrambled; Dharmacon) or siRNA to *KAP1* (Dharmacon). Transfected cells were treated with NaB 24 hours later and harvested at indicated times post-treatment for immunoblotting with indicated antibodies. Numbers below bands indicate relative amounts of ZEBRA after normalization to β-actin.
(TIF)

S2 Fig NaB does not induce phosphorylation of KAP1 in an EBV-uninfected B lymphoma cell line. BJAB cells were treated with NaB for 24 hours or etoposide (Etp) for 4 hours. Cell lysates were subjected to immunoblotting for p-S824 KAP1 and total KAP1 levels.
(TIF)
