# Supplementary figures and images for: Chloroquine triggers Epstein-Barr virus replication through phosphorylation of KAP1/TRIM28 in Burkitt lymphoma cells
Source: PLoS Pathog. 2017 Mar 1;13(3):e1006249. doi: 10.1371/journal.ppat.1006249 (PMC5348047; doi:10.1371/journal.ppat.1006249)

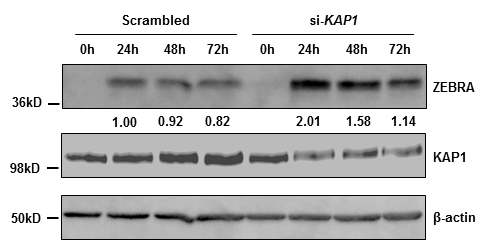

Supplement: S1 Fig — HH514-16 Burkitt lymphoma (BL) cells were transfected with a non-targeting control siRNA (scrambled; Dharmacon) or siRNA to KAP1 (Dharmacon). Transfected cells were treated with NaB 24 hours later and harvested at indicated times post-treatment for immunoblotting with indicated antibodies. Numbers below bands indicate relative amounts of ZEBRA after normalization to β-actin. (TIF) [file ppat.1006249.s001.tif]

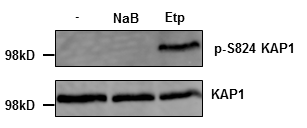

Supplement: S2 Fig — BJAB cells were treated with NaB for 24 hours or etoposide (Etp) for 4 hours. Cell lysates were subjected to immunoblotting for p-S824 KAP1 and total KAP1 levels. (TIF) [file ppat.1006249.s002.tif]
